# A Long-Time Series Forecast Method for Wind Turbine Blade Strain with Incremental Bi-LSTM Learning

**DOI:** 10.3390/s25133898

**Published:** 2025-06-23

**Authors:** Bingkai Wang, Wenlei Sun, Hongwei Wang

**Affiliations:** School of Mechanical Engineering, Xinjiang University, Urumqi 830047, China; wangbk@stu.xju.edu.cn (B.W.); wanghongwei@stu.xju.edu.cn (H.W.)

**Keywords:** Bi-LSTM, incremental learning, long-time series forecast, strain, wind turbine blade

## Abstract

This article presents a novel incremental forecast method to address the challenges in long-time strain status prediction for a wind turbine blade (WTB) under wind loading. Taking strain as the key indicator of structural health, a mathematical model is established to characterize the long-time series forecast forecasting process. Based on the Bi-directional Long Short-Term Memory (Bi-LSTM) framework, the proposed method incorporates incremental learning via an error-supervised feedback mechanism, enabling the dynamic self-updating of the model parameters. The experience replay and elastic weight consolidation are integrated to further enhance the prediction accuracy. Ultimately, the experimental results demonstrate that the proposed incremental forecast method achieves a 24% and 4.6% improvement in accuracy over the Bi-LSTM and Transformer, respectively. This research not only provides an effective solution for long-time prediction of WTB health but also offers a novel technical framework and theoretical foundation for long-time series forecasting.

## 1. Introduction

Wind energy, as the most popular renewable energy source, is gradually replacing fossil fuels [1]. Projections indicate an anticipated increase of 1210 GW in installed capacity by 2030, potentially supplying over 36% of global electricity demand [2,3]. Therefore, the research on the technologies related to wind turbine has attracted much attention [4]. As the most critical component of the wind turbine, the wind turbine blade (WTB) has undergone substantial development, with current designs reaching 90–150 m and exceeding 30 t [5]. Such a huge size and weight create significant health concerns. Furthermore, continuous exposure to harsh environmental conditions subjects WTBs to alternating loads and surface erosion, increasing vulnerability to multiple failure modes including surface cracking, abrasion, icing, and catastrophic breakage. According to the research by Wen [6], failure of the WTB represents the primary cause of downtime, accounting for 34%. In addition, the maintenance cost of WTBs accounts for about 20% of the total equipment maintenance costs [7,8]. Therefore, it is important to accurately forecast the health status of the WTB as early as possible in order to make reasonable maintenance decisions in advance.

At first, the structural response analysis and fatigue damage mechanisms are employed to forecast the health status of the WTB. For example, Meng [9] analyzes the fatigue life of a WTB based on anisotropic beam model and stress-based fatigue method. Caous [10] uses ply scale damage model to evaluate WTB fatigue life. Li [11] proposes a method for a combined high and low cycle fatigue life prediction model based on Miner’s rule. How-ever, the accuracy of these forecast methods is compromised by environmental variability.

Subsequently, the method based on data analysis have received lots of attention. The commonly employed carrier for WTB health information is the SCADA data. For example, Lu [12] proposes a small-sample forecast method via a combination of multiple neural networks based on SCADA data. Antoine [13] evaluates the WTB damage under multiple conditions based on SCADA data. But the information reflected via SCADA is too vague for large-scale WTBs because it is low-frequency and indirect. Strain is the physical quantity that most directly reflects the status of a structure, and the techniques based on strain detection have been widely employed to monitor the health status of WTBs. For example, the research studies [14,15,16] evaluated the performance of WTBs via monitoring strain changes. However, the time course of WTB strain signals tends to exhibit nonlinear, periodic and unstable characteristics during the degradation process of WTBs. Therefore, how to find out the change rule of data characteristics through the highly complex strain information is the most difficult problem in the prediction of the health status of WTBs.

In order to address the above problem, the machine learning and intelligent algorithms are gradually employed to forecast the health status of WTBs. For example, Deng [17] forecasts the deformation of WTBs via combining of machine learning and mechanism analysis. Liu [18] employs deep learning to forecast the stiffness of the WTB. Choe [19] pro-poses a sequence-based modeling for structural damage detection of the WTB using Long Short-Term Memory and Gated Recurrent Unit neural networks. Although these methods can accurately forecast the short-term health status of a WTB, they perform poorly in the long-term. Unfortunately, the demand for forecasting the future health status of WTBs as early as possible poses a serious challenge to long-time series forecast method.

This study proposes a long-time series forecast method in order to improve the prediction accuracy of strain. Compared to other networks currently, the Bi-LSTM network is the most mature in solving time series problems and it receives the widest application because of its stable performance. Therefore, the Bi-LSTM network is chosen as the basic network for the long-time series forecast method proposed in this article, and the incremental learning is adopted to improve the long-time forecasting performance.

## 2. Theoretical Background

### 2.1. Problem Statement

The health degradation of a WTB is a gradual process over time. Then, the forecast of the health status of a WTB via strain data is a time series forecast problem. The idea of using neural networks to solve the time series prediction problem is originally proposed by Weigend in *International Journal of Neural Systems* [20]. In this idea, the input to the model is defined as a historical vector in a sliding time window, and the output is defined as a future value. A time series with length L and dimension *n* can be recorded as historical data $X=(x_{1},x_{2},\ldots ,x_{L})\in R^{L\times n}$, and a movable retrospect window with length *e* is denoted as $X_{i,e}=(x_{i-e+1},x_{i-e+2},\ldots ,x_{i})$. It is the goal of time series forecast to estimate the series in the future period after time *i* based on the latent regularity mined via the retrospect window from the historical data before time *i*. The length of this future period is known as the forecast window and is denoted as H. Then the mathematical expression of the time series forecast is

(1) $$\begin{array}{cc} forecast\;values & =f_{\omega }(input\;values)\\ \left(x_{i+1},x_{i+2},\ldots x_{i+H}\right) & =f_{\omega }(X_{i,e})\\ \left(x_{i+1},x_{i+2},\ldots x_{i+H}\right) & =f_{\omega }(x_{i-e+1},x_{i-e+2},\ldots x_{i}) \end{array}$$

where ω is the parameters of the forecast model. It is required to minimize the error between the forecast value and the true value throughout the forecasting process.

**Figure 1:**
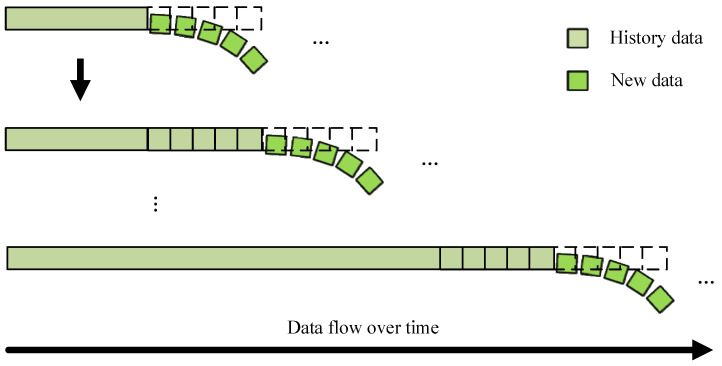
Online time series forecast task.

In the practical applications, the data arrive sequentially as continuous streams, as shown in Figure 1. The online time series forecasting task necessitates incremental learning new knowledge from a continuous input data stream, and the training and prediction of the model occur alternated. Since the current health status exhibits temporal dependencies on previous states, comprehensive historical data must be considered when predicting future conditions. However, historical data are constantly increasing, and the huge amount of data will be accompanied by high computing cost and computational time. Consequently, another tricky issue in forecasting the health of WTBs is how to reduce memory occupation while ensuring high accuracy.

### 2.2. Bi-LSTM Networks

The LSTM network, originally proposed by Hochreiter in *Neural Computation* [21], has unique capabilities which make it very suitable for time series forecasting problems. The LSTM network allows the entire network to have longer feature memory via designing a unique gate structure in every cell. In addition, the unique gate structure enables the LSTM network to discard the invalid components of the transmission data from previous neuronal and retain useful information effectively. Thus, the LSTM network shows good stability in time series prediction. In order to improve the forecast accuracy, the Bi-LSTM network is adopted as the fundamental framework of the model to forecast the health status of WTBs.

**Figure 2:**
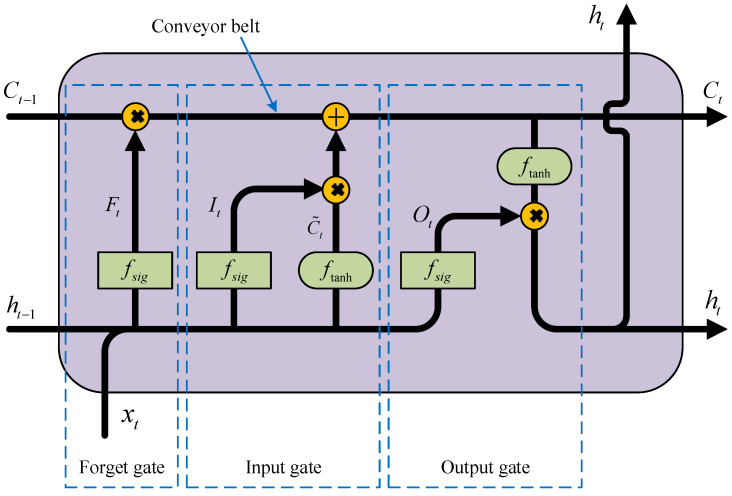
Cell structure of LSTM model.

The LSTM network is essentially an improved RNN. As shown in Figure 2, the cellular structure inside the LSTM network mainly consists of the conveyor belt, forget gate, input gate and output gate [22].

The method of updating the cellular status of the LSTM network can be expressed as follows in Equation (2):

(2) $$\left\{\begin{array}{l} F_{t}=f_{sig}(W_{f}\cdot [h_{t-1},x_{t}]+b_{f})\\ I_{t}=f_{sig}(W_{i}\cdot [h_{t-1},x_{t}]+b_{i})\\ {\tilde{C}}_{t}=f_{\tanh}(W_{c}\cdot [h_{t-1},x_{t}]+b_{c})\\ C_{t}=F_{t}C_{t-1}+I_{t}{\tilde{C}}_{t}\\ O_{t}=f_{sig}(W_{o}\cdot [h_{t-1},x_{t}]+b_{o})\\ h_{t}=O_{t}f_{\tanh}(C_{t}) \end{array}\right.$$

where *F_t_*, *I_t_*, ${\tilde{C}}_{t}$, *C_t_*, *O_t_*, *h_t_* and *x_t_* are, respectively, forget gate vector, input gate vector, new value vector, status vector, output gate vector, output vector and input vector. *W* is the weight coefficients matrix, *b* is the bias coefficient vector, and *f* is the activation function. It is worth noting that all parameter matrices in the model are independent of each other and need to be trained via back propagation.

**Figure 3:**
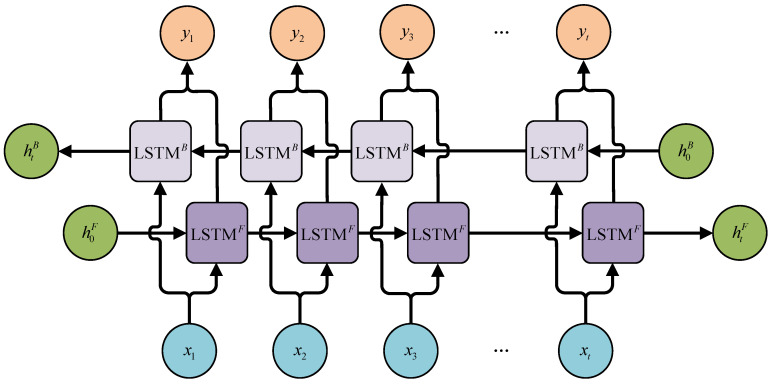
Structure of Bi-LSTM networks.

In order to further improve the memory ability, the Bi-LSTM architecture, initially introduced by Graves in *Neural Networks* [23], incorporates an additional reverse direction propagation path to the classical LSTM framework, as shown in Figure 3.

The forward and backward propagation paths operate independently with distinct model parameters. In the forward path, the output $h_{t}^{F}$ gradually forgets the earliest inputs at the left. Conversely, the output $h_{t}^{B}$ of the backward propagation path gradually forgets the earliest inputs at the right. The forward propagation and back propagation for updating the cellular status of the Bi-LSTM network can be expressed as Equation (3) and Equation (4), respectively.

(3) $$\left\{\begin{array}{l} {\vec{F}}_{t}=f_{sig}(W_{f}\cdot [{\vec{h}}_{t-1},x_{t}]+b_{f})\\ {\vec{I}}_{t}=f_{sig}(W_{i}\cdot [{\vec{h}}_{t-1},x_{t}]+b_{i})\\ {\vec{\tilde{C}}}_{t}=f_{\tanh}(W_{c}\cdot [{\vec{h}}_{t-1},x_{t}]+b_{c})\\ {\vec{C}}_{t}={\vec{F}}_{t}{\vec{C}}_{t-1}+{\vec{I}}_{t}{\vec{\tilde{C}}}_{t}\\ {\vec{O}}_{t}=f_{sig}(W_{o}\cdot [{\vec{h}}_{t-1},x_{t}]+b_{o})\\ {\vec{h}}_{t}={\vec{O}}_{t}f_{\tanh}({\vec{C}}_{t}) \end{array}\right.$$

(4) $$\left\{\begin{array}{l} {\overset{\leftarrow }{F}}_{t}=f_{sig}(W_{f}\cdot [{\overset{\leftarrow }{h}}_{t-1},x_{t}]+b_{f})\\ {\overset{\leftarrow }{I}}_{t}=f_{sig}(W_{i}\cdot [{\overset{\leftarrow }{h}}_{t-1},x_{t}]+b_{i})\\ {\overset{\leftarrow }{\tilde{C}}}_{t}=f_{\tanh}(W_{c}\cdot [{\overset{\leftarrow }{h}}_{t-1},x_{t}]+b_{c})\\ {\overset{\leftarrow }{C}}_{t}={\overset{\leftarrow }{F}}_{t}{\overset{\leftarrow }{C}}_{t-1}+{\overset{\leftarrow }{I}}_{t}{\overset{\leftarrow }{\tilde{C}}}_{t}\\ {\overset{\leftarrow }{O}}_{t}=f_{sig}(W_{o}\cdot [{\overset{\leftarrow }{h}}_{t-1},x_{t}]+b_{o})\\ {\overset{\leftarrow }{h}}_{t}={\overset{\leftarrow }{O}}_{t}f_{\tanh}({\overset{\leftarrow }{C}}_{t}) \end{array}\right.$$

The output is obtained via combining $h_{t}^{F}$ and $h_{t}^{B}$, as shown in Equation (5). Thus, the problem of forgetting the earliest information is solved. It is noteworthy that the combination of $h_{t}^{F}$ and $h_{t}^{B}$ is achieved via vector concatenation. The dimension of the new vector $H_{t}$ is the sum of the dimensions of $h_{t}^{F}$ and $h_{t}^{B}$.

(5) $$\left\{\begin{array}{l} h_{t}^{F}=LSTM^{F}(h,x,c)\\ h_{t}^{B}=LSTM^{B}(h,x,c)\\ H_{t}=[h_{t}^{F},h_{t}^{B}] \end{array}\right.$$

### 2.3. Incremental Learning

In the engineering of WTB health status prediction, the target often occupies a long-time span in the future. Forecasting the future strain as early and accurately as possible is of great engineering importance for the predictive operation and maintenance of WTBs. However, it is still limited in a short and fixed time span to accurately forecast the future data via Bi-LSTM network. Therefore, this research introduces an incremental learning strategy to construct a forecast model that can self-update dynamically in order to enhance the long-time forecasting performance based on the Bi-LSTM network.

**Figure 4:**
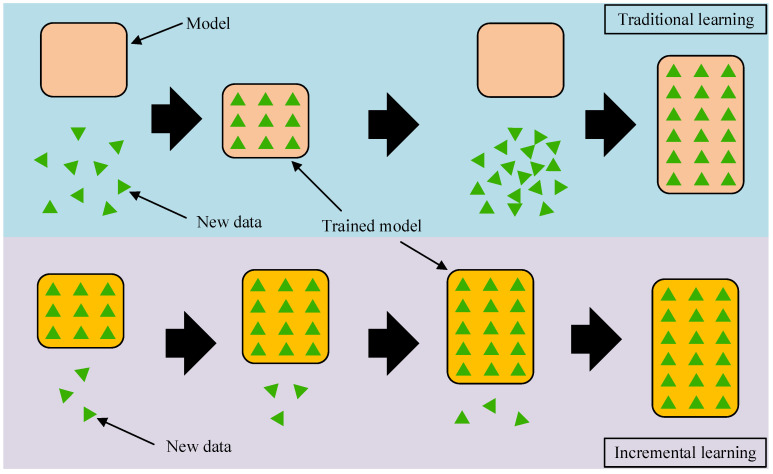
The difference between incremental learning and traditional learning.

Incremental learning [24,25], also referred to as continuous learning, is a learning strategy designed for sequentially arriving data. Consequently, the forecast model can gradually adapt to the changes of the external information environment. Within neural network systems, incremental learning enables continuous new knowledge learned from a data stream while preserving memory learned previously. As the new data become available over time, the pre-trained forecast model learns the new knowledge in real time under this strategy. In the process of incremental learning, the prior knowledge is conducive to improving the learning efficiency for new knowledge, while the new data are conducive to improving the forecasting performance in the new information environment. Therefore, incremental learning has the following advantages compared to traditional learning strategies: (1) Improve prediction accuracy via continuously and dynamically optimizing model parameters based on new data. (2) Avoid retraining the model from scratch using a large amount of historical data, and instead utilize a small amount of new data to reduce and memory occupation. Figure 4 illustrates the difference between incremental and traditional learning strategies.

The primary objective of incremental learning is to update the model parameters via gradually accepting new data samples without retraining the entire model. Particularly in the prediction of WTB strain, incremental learning permits the forecast model to automatically adapt the model parameters according to accumulating strain data.

## 3. Proposed Incremental Health Status Forecast Method

### 3.1. Overall Architecture of Method

**Figure 5:**
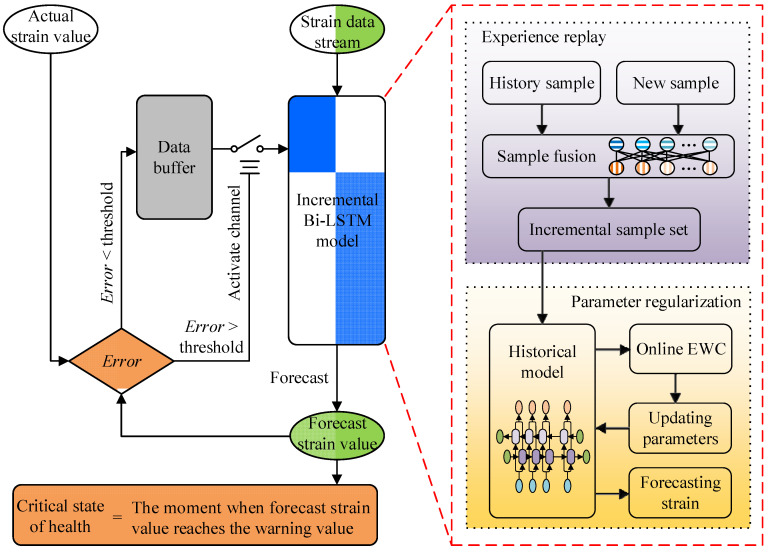
The overall process of incremental health status forecasting.

In order to address the two critical challenges mentioned in Section 2.1, this research presents an incremental forecast method based on Bi-LSTM networks and incremental learning theory. The complete workflow of this incremental forecast method is shown in Figure 5.

The WTB strain data are continuously acquired via sensors, forming a data stream that serves as input to the incremental Bi-LSTM model. This model is the computational core in the health status forecast method. It is worth noting that the incremental Bi-LSTM model at this point is not in a fully initialized state, but has been trained previously via historical data. After the strain data stream of the current period passes through the model, the model generates the WTB strain information for the future period and output the strain forecast value. When the strain forecast value reaches the warning threshold, it is identified that fatigue failure will occur at that moment. This warning threshold is commonly determined via fatigue testing of the WTB and historical experience. Usually, a WTB is considered to fail when its stiffness degrades by more than 10% [26]. Therefore, the warning threshold of strain can be set to 110% of the initial strain at the same wind speed.

Over time, the measured value at future moments corresponding to the forecast value will be measured via the strain sensors installed on the WTB. The measured value can be employed to check the accuracy of the forecast value. The process is a biased estimation problem based on small sample, so this research adopts the root mean square error (RMSE) to evaluate the deviation between the forecast value and the actual value. The formula for RMSE is as Equation (6),

(6) $$\mathrm{RMSE}=\sqrt{\frac{1}{n}\sum_{i=1}^{n}{\left(y_{i}-{\hat{y}}_{i}\right)}^{2}}$$

where ${\hat{y}}_{i}$ is the *i*th forecast value, $y_{i}$ is the *i*th actual value. If the RMSE is less than the threshold, the forecast value is considered accurate and credible, and the new measured data are subsequently stored in a data buffer. According to engineering experience, 10% of the mean of the training dataset is selected as the error threshold in this article. It is important to emphasize that the threshold for deviation judgments is not the same one as the warning threshold for critical state of health.

**Figure 6:**
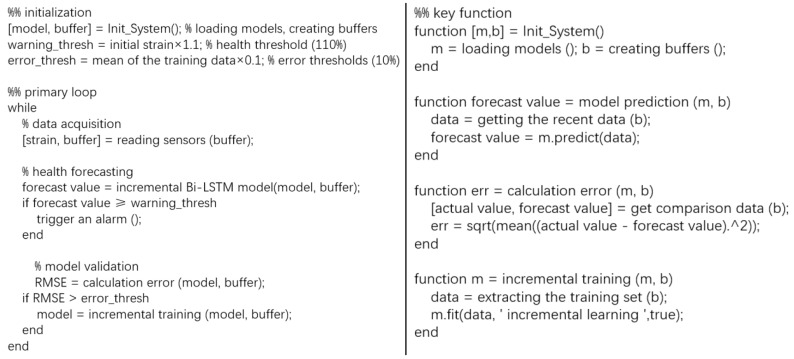
The pseudocode for the workflow.

A normal-off gate is designed to regulate the data flow between the data buffer and the incremental Bi-LSTM model. As the new data arrive continually, errors continue to accumulate and the deviation between forecast value and the actual value will become larger. When the RMSE is greater than the threshold, the forecast value is considered to be unreliable. Then, the channel between the data buffer and the incremental Bi-LSTM model is activated. The new data stored in the data buffer are fed into the incremental Bi-LSTM model to incrementally train the historical model. The design of the normal-off gate structure with error judgment and the data buffer realizes the adaptive updating of the incremental Bi-LSTM model. The updated forecast model can continuously output accurate and credible forecast values. The pseudocode for the forecast process is shown in Figure 6.

The incremental Bi-LSTM model, as the key component of the WTB health status forecast method, is mainly composed of two modules: the experience replay module and the parameter regularization module. The experience replay module generates an incremental sample set via feature relevance computation for subsequent incremental training. The parameter regularization module adopts the elastic weight consolidation (EWC) method to regulate the parameter updates of the historical model during incremental training. The updated forecast model has the historical priori knowledge while adapting to the new information environment and can be employed for forecasting subsequent strain value. The experience replay module and the parameter regularization module are described in detail in the following sections, respectively.

### 3.2. Incremental Learning Based on Experience Replay

**Figure 7:**
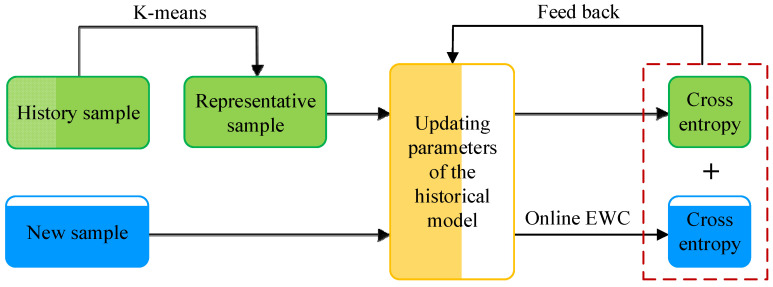
Principles of experience replay technology.

With the degradation of the WTB health status, the characteristics of new strain data progressively diverge from historical patterns. Then, historical knowledge will be covered when the model learns new knowledge via the new data. This is the key challenge to incremental learning, namely catastrophic forgetting. The lack of historical knowledge can degrade the forecasting performance of the model. In order to mitigate catastrophic for-getting, this research integrates the experience replay [27] technology into the incremental learning. As shown in Figure 7, the core idea of the experience replay technology is to add historical samples to the new samples, forming an incremental sample set. In addition to the new sample information, the incremental sample set also carries the characteristics of the historical task. In this way, the historical samples participate in the incremental training of the model, enabling to effectively prevent forgetting previous knowledge. However, incorporating the complete historical dataset would incur prohibitive computational cost and increase risks of overfitting or convergence difficulties. Consequently, selectively incorporating representative historical samples into the incremental sample set presents a practical approach.

A new problem arises as how to store more important samples in the limited memory space. Typically, classical experience replay technology uses random sampling with uniform probability to select the samples from a large number of historical samples. However, the method ignores the differential contributions of individual historical samples to the model performance. In this research, the K-Means clustering algorithm is employed to select representative samples from a large number of historical samples because of its computational efficiency and good clustering results. The K-Means clustering algorithm firstly divides the historical samples into several feature clusters based on the distance between the feature vectors. The distance of each sample point in the dataset from each cluster center is calculated according to Equation (7), and each sample point is assigned to the cluster center with the closest distance;

(7) $$dist(x,c_{i})=\sqrt{\sum_{j=1}^{d}{\left(x_{j}-c_{ij}\right)}^{2}}$$

where *x* is sample point, *c_i_* is the cluster center, *d* is the dimensions of data, *x_j_* and *c_ij_* are the values on *j*, respectively. Then, the new clustering center is recalculated based on each feature cluster as Equation (8):

(8) $$c_{i}=\frac{1}{\left|S_{i}\right|}\sum_{x\in S_{i}}x$$

where *S_i_* is the set of sample points for the *i*th cluster.

Subsequently, some samples closest to the center of each feature cluster are selected as representative samples. These representative samples carry historical knowledge and reflect the overall characteristics of the historical sample. The representative samples are combined with new samples to form the incremental sample set.

The cross entropy is usually employed as the loss function for training historical models on incremental sample sets, providing the optimization objective for updating of forecast model parameters. However, the imbalance in the number of new and representative samples induces significant gradient instability, which can cause drastic changes in the model parameters. In order to address this issue, the losses of the representative and new samples are calculated separately. This separation eliminates direct comparison between historical and new samples. The final loss function is defined as the sum of the cross entropies of the representative and new samples, as shown in Equation (9);

(9) $$L_{model}(\theta )=L_{his}(\theta )+L_{new}(\theta )$$

where θ is the model parameters of the current status, and $L_{model}$, $L_{his}$, and $L_{new}$ are the final loss of the model, the loss of the representative sample and the loss of the new sample, respectively.

### 3.3. Parameters Regularization via Elastic Weight Consolidation

Although the representative samples mentioned above are effective in expressing the overall characteristics of historical samples, some samples located at the boundaries of feature clusters are equally crucial to historical knowledge. It may also affect the fore-casting accuracy to ignore these boundary samples. Unfortunately, the identification of boundary samples is difficult and computationally expensive. Therefore, in addition to optimizing the selection of historical sample, other technical methods need to be incorporated to further improve the learning performance of the model.

The fundamental mechanism by which training samples influence model performance lies in the differential informational contribution of sample features to parameter updates. Certain parameters exhibit high sensitivity to variations in the sample. Conversely, other parameters have less information from the historical samples and the knowledge already learned cannot be covered when updating these parameters. Thus, the problem of forgetting knowledge can be mitigated via imposing regularization on the highly sensitive parameters. Based on this idea, the EWC is incorporated into the training process on new samples to compensate for the neglect of boundary samples, as shown in Figure 6.

The EWC [28] employs the Fisher information matrix to impose regularization on the updating of model parameters based on varying importance, because the Fisher information matrix is a good measure of the sensitivity of the model parameters. Specifically, the EWC redefines the loss of a new sample as the sum of the cross entropy of the new sample and the total deviation of all the parameters of the model, as shown in Equation (10);

(10) $$L_{new}(\theta )=L_{new}^{*}(\theta )+\frac{\lambda }{2}\sum_{i}\Omega_{i}{\left(\theta_{i}^{*}-\theta_{i}\right)}^{2}$$

where $L_{new}^{*}(\theta )$ is the cross entropy of the new sample, which is numerically equal to that in Equation (9). $\theta_{i}$ and $\theta_{i}^{*}$ are the model parameters before and after the update, respectively. $\Omega_{i}$ represents the importance of the model parameters. λ is the regulating factor.

The second term in Equation (10) is the regularization term demonstrating that the parameter with high weight should be close before and after updating in order to minimize the overall loss. The knowledge learned from the historical samples in the model is retained via the regularization.

The regularization term can be obtained based on Bayesian theory. The strain data of the WTB is noted as $D=\left\{D_{his},D_{new}\right\}$, where *D_his_* and *D_new_* are historical and new data, respectively. In the Bayesian perspective, the goal of training the model parameters changes from minimizing the loss function to maximizing the posterior probability of the model parameters $\log p\left(\theta |D\right)$. Then, the parameters of the model should obey the posterior probability distribution:

(11) $$\begin{array}{ll} \log p\left(\theta |D\right) & =\log p\left(D_{his},D_{new}|\theta \right)+\log p\left(\theta \right)-\log p\left(D_{his},D_{new}\right)\\ & =\log p\left(D_{his}|\theta \right)+\log p\left(D_{new}|\theta \right)+\log p\left(\theta \right)-\log p\left(D_{his}\right)-\log p\left(D_{new}\right)\\ & =\log p\left(D_{new}|\theta \right)+\log p\left(\theta |D_{his}\right)-\log p\left(D_{new}\right) \end{array}$$

In Equation (11), the first log probability $\log p\left(D_{new}|\theta \right)$ can be viewed as the negative of the loss of the new sample, i.e., $-L_{new}^{*}(\theta )$ in Equation (10). The third term $\log p\left(D_{new}\right)$ is a constant independent of the parameters and can be disregarded. The second term $\log p\left(\theta |D_{his}\right)$ is written into functional form as Equation (12);

(12) $$p\left(\theta |D_{his}\right)=\frac{1}{Z}f(\theta )$$

where *Z* is normalization factor. The Taylor expansion of its likelihood function is then performed as Equation (13);

(13) $$\ln f(\theta )=\ln f(\theta^{*})+J_{\ln f}(\theta^{*})(\theta -\theta^{*})+\frac{1}{2}{\left(\theta -\theta^{*}\right)}^{T}H_{\ln f}(\theta^{*})(\theta -\theta^{*})+R_{n}(\theta )$$

where $\theta_{i}^{*}$ is the parameter trained previously, $H_{\ln f}$ is the Hessian matrix. The first order terms and terms above the third order are approximated as 0 because the loss is small in the model trained. Thus, only the second order and zero order terms are retained as Equation (14).

(14) $$\ln f(\theta )\approx \ln f(\theta^{*})-\frac{1}{2}{\left(\theta -\theta^{*}\right)}^{T}F_{\ln f}(\theta^{*})(\theta -\theta^{*})\;F_{\ln f}=-H_{\ln f}(\theta^{*})$$

Since the Fisher information matrix is the negative of the expectation of the Hessian matrix of the log-likelihood function, the Fisher information matrix is used instead of the Hessian matrix for convenience of calculation. Taking the index of Equation (14) yields Equation (15).

(15) $$f(\theta )\approx \frac{1}{Z}f(\theta^{*})e^{-\frac{1}{2}{\left(\theta -\theta^{*}\right)}^{T}F_{\ln f}(\theta^{*})(\theta -\theta^{*})}$$

Therefore, the $\log p\left(\theta |D_{his}\right)$ can be regarded as a Gaussian distribution obeying $\mu =\theta_{i}$, $\frac{1}{\sigma^{2}}=F_{\ln f(\theta )}$. Then, the maximization of $\log p\left(\theta |D_{his}\right)$ is equivalent to maximization of $-\frac{1}{2}{\left(\theta -\theta^{*}\right)}^{T}F_{\ln f}(\theta^{*})(\theta -\theta^{*})$, and Equation (10) can ultimately be ex-pressed as

(16) $$\begin{array}{c} L_{new}(\theta )=L_{new}^{*}(\theta )+\frac{\lambda }{2}\sum_{i}F_{i}{\left(\theta_{i}^{*}-\theta_{i}\right)}^{2}\\ F_{i}={\left(\frac{\partial L_{new}^{*}(\theta_{i}^{*})}{\partial \theta_{i}^{*}}\right)}^{2} \end{array}$$

It should be emphasized that health status prediction of the WTB is an online time series forecasting task. A new training task is initiated upon activation of the normal-off gate described in Figure 5. As the number of history tasks continues to increase, the EWC calculates a regularization term for all history tasks. Then, the regularization term grows linearly with the data flow, resulting in a large computational cost. Since each new task is trained via a regularization term to the previous history task, the regularization term of the previous task can contain the constraints of all previous history tasks. Then, the weighted sum of the Fisher information matrix of all historical tasks is performed, and only the parameters of the previous one task need to be regularized. Therefore, the online form of Equation (16) can be expressed as

(17) $$L_{new}(\theta )=L_{new}^{*}(\theta )+\frac{1}{2}\sum_{i}\left(\sum_{t<new}\lambda_{t}F_{t,i}\right){\left(\theta_{i}^{*}-\theta_{new-1,i}\right)}^{2}$$

Equation (17) can retain the information characteristics of historical samples as much as possible, so as to achieve more accurate online prediction for the health status of the WTB.

## 4. Experiments and Data Analysis

### 4.1. Load Scheme Design

In order to validate the proposed long-time series forecast method, the WTB strain measurement experiment is designed in this research. The strain data of the WTB under simulated wind load in experimental conditions is collected, and is used as the time series dataset to validate the proposed method.

**Figure 8:**
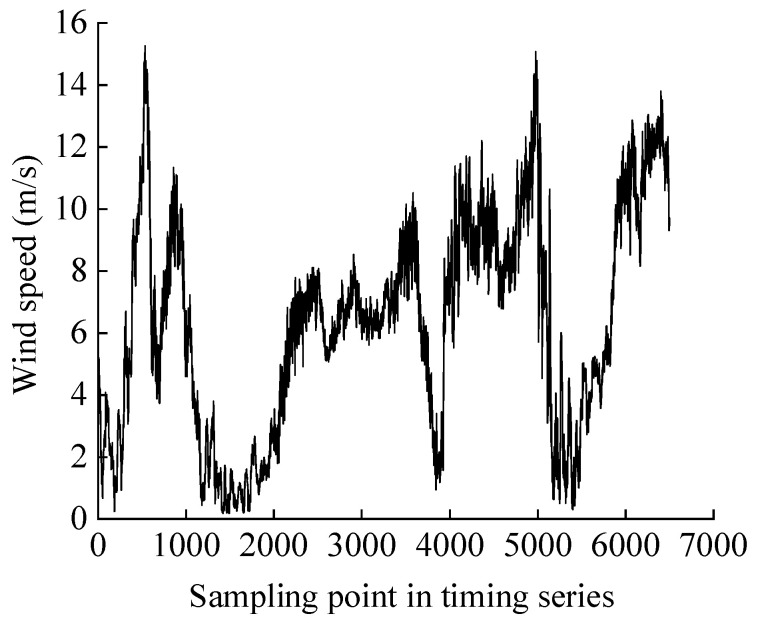
Wind speed data.

This research collects the wind speed data spanning 168 h in a week from a wind farm in Darbancheng, Xinjiang, in order to guide the load scheme design. Each wind speed value is sampled as the average wind speed value during the current 1 min time period. Invalid data points in the data are eliminated, such as exceeding the maximum operating wind speed. The final wind speed data time series contains 6500 wind speed sampling points, as shown in Figure 8.

Ideally, wind speed data should be set as a load spectrum and then loaded on the WTB via a wind tunnel test. However, the large-scale wind tunnel experimental conditions are often demanding and costly. Consequently, researchers often design concentrated loads instead of wind loads in the WTB tests, as demonstrated in other peer studies [29]. The experiments in this research require precise control of the loading history based on the wind speed data. Because it has the advantage of being able to respond accurately and quickly to irregular fluctuations in load, the forced displacement loading method is finally selected to simulate the wind loads after comprehensive consideration.

Furthermore, given that each wind speed sampling point represents the average value in one minute, the corresponding load should not be an instantaneous load, but a sustained load over a period of time. Therefore, in order to simulate the actual wind load, the loading of each wind speed sampling point should continue for a period of time be-fore transitioning to the subsequent wind speed sampling point. Notably, even if the wind speed is constant in actual conditions, the WTB does not keep a static deformation, but vibrate repeatedly within the range of the maximum deformation corresponding to the wind speed (only the flap direction is considered in this research). Accordingly, the WTB is designed to repeatedly cycle within a range of deformation corresponding to each wind speed sampling point. The duration is defined as the flap-period of a single wind speed sampling point. The repeated cycles during each flap-period are defined as flap-cycles. Then, it proceeds to the next flap-period of wind speed sampling points until the loading history of all wind speed sampling points is completed.

### 4.2. Data Acquisition

**Figure 9:**
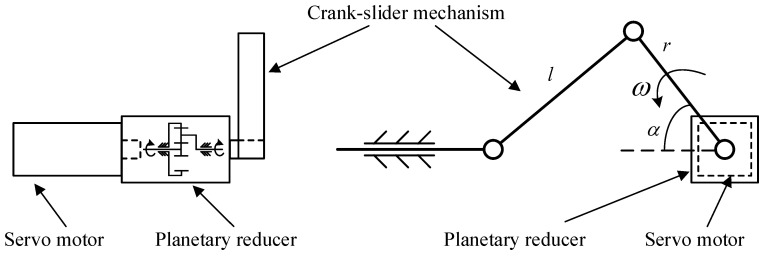
The experimental loading equipment.

After designing the loading scheme, this research adopts a servo motor as the controllable power output, a crank-slider mechanism as the load transfer mechanism, and a microcontroller to control load fitting the wind speed data. The overall schematic of the experimental loading equipment is shown in Figure 9.

In order to ensure smoother operation of the whole equipment, a one-stage planetary reducer is installed between the servo motor and the crank to increase the input torque. The crank, as the original moving part of the load transmission mechanism, converts the initial circular motion into a linear reciprocating motion via the crank slider mechanism. The slider is fixed on the WTB via the clamp to realize the forced displacement loading on the WTB. The displacement x of the loading mechanism can be determined by Equation (18);

(18) $$x=r\cos\alpha +\sqrt{l^{2}-r^{2}\sin^{2}\alpha }$$

where α is crank rotation angle, α=ωt. ω and *t* are the crank angular velocity and time, respectively, *r* and *l* are the length of crank and connecting rods, respectively.

A 2.4 m scaled WTB is used for strain measurement experiment. The unidirectional deformation value of the WTB corresponding to the maximum wind speed data is 7.21 cm, which is calculated via the fluid–structure coupling simulation of rotating impellers in finite element software. Accordingly, the crank length is set to 7.21 cm, then the maximum displacement stroke is 14.42 cm. The crank angulars corresponding to the maximum and minimum limit positions are 0° and 180°, respectively. The displacements corresponding to different wind speed are fitted based on the deformation values of the WTB obtained from the simulation, then the crank angular corresponding to a certain of wind speed is calculated via Equation (18). During the experiment, the initial position of the crank was set at 90° and the flap-period corresponding to each wind speed sampling point is set to 3 s. The “90° to maximum angle to 90° to minimum angle to 90°” is a flap-cycle lasting 1 s, and the crank undergoes a total of 3 cycles in each flap-period.

According to the wind speed course shown in Figure 7 and the above loading scheme, a control program for motor shaft rotation angle is written and embedded into the microcontroller to control the servo motor to complete the entire loading course. Notably, the crank angle is the motor output shaft value after deceleration via the reducer.

**Figure 10:**
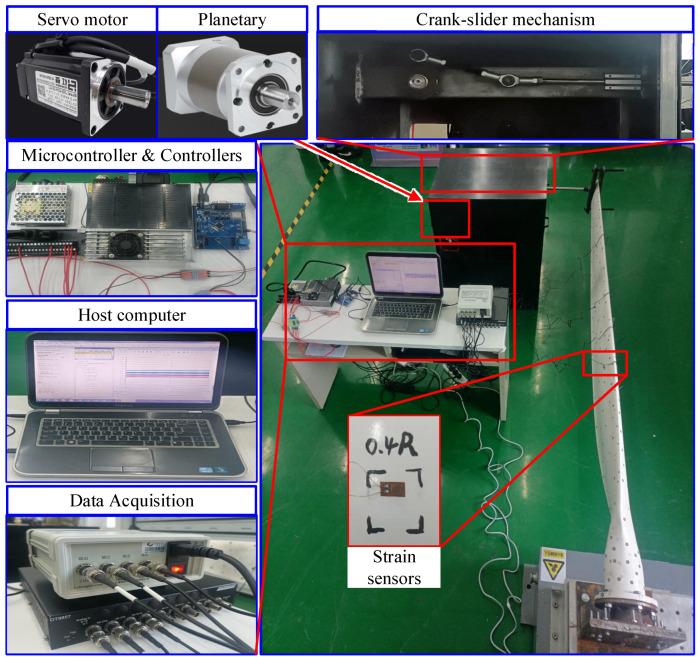
The random load WTB strain measurement experiments.

The servo motor used in the experiment is a Hongsen AC servo motor, and the model is JXE-751K30-BJ-B (made in China), with a power of 750 W and a rated output torque of 2.4 Nm. The reducer is a First single-stage planetary reducer, and the model is WLF080 (made in China), with a gear reduction ratio of 10, maximum output torque of 40 Nm, and maximum input speed of 6000 rpm. The crank-slider mechanism is designed and machined in-house. The WTB is fixed on the experimental fixture with flanges in the form of end restraints. The experimental site for strain status detection of the WTB under simulated wind load is shown in Figure 10.

The entire length of the WTB is defined as R. Three strain sensors, which are employed to measure and record the WTB strain data, are installed at 0.4R, 0.5R, and 0.6R along the WTB span direction with epoxy resin adhesive, and numbered as strain sensor 1, strain sensor 2, and strain sensor 3, respectively. These positions have received attention in many studies [30,31]. The sensors are resistance strain gauges and Table 1 describes the strain sensors models and characteristics. The strain data were sampled at a frequency of 1000 Hz.

In addition, the host computer used in the experiment is a personal laptop (PC) with Windows 7 64-bit operating system and a CPU of Intel Core i5-3230M. The data acquisition model is DT9857 (made by Measurement Computing Corporation in USA) and the program accompanying Quick DAQ (DAQami 3.2) is used as software. Model calculations are done under MATLAB R2023b software.

### 4.3. Signal Preprocessing and Analysis

**Figure 11:**
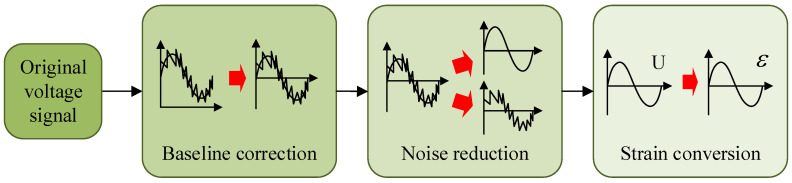
The signal preprocessing flow.

The preprocessing flow of signal is shown in Figure 11. The data acquired via the sensors are a one-dimensional time series of voltage values. Firstly, the direct current drift in original signal is removed via mean subtraction in order to correct the baseline.

The signal contains unavoidable noise, which mainly originate from the current interference inherent within the system, such as switching power supply noise, grid harmonics, and inductive coupling. The frequency of the grid harmonics is 50 Hz, which is the lowest of the above noises. The frequency of the WTB strain is below 10 Hz; therefore, a low-pass filter can be used for noise reduction. The 1st order Butterworth filter is adopted because it preserves the amplitude accuracy better and avoids phase delays. Typically, the cutoff frequency is greater than twice the target frequency and less than the noise frequency. Four frequencies, 25, 30, 35, and 40 Hz, are compared, and the best 35 Hz is selected as the cutoff frequency.

After the noise reduction process, every voltage value is converted to strain value according to Equation (19);

(19) $$\varepsilon =\frac{4\times U\times 10^{6}}{K\times 5.02\times 1\times 405.321}$$

where *U* is voltage value and K is sensitivity coefficient of sensors.

**Figure 12:**
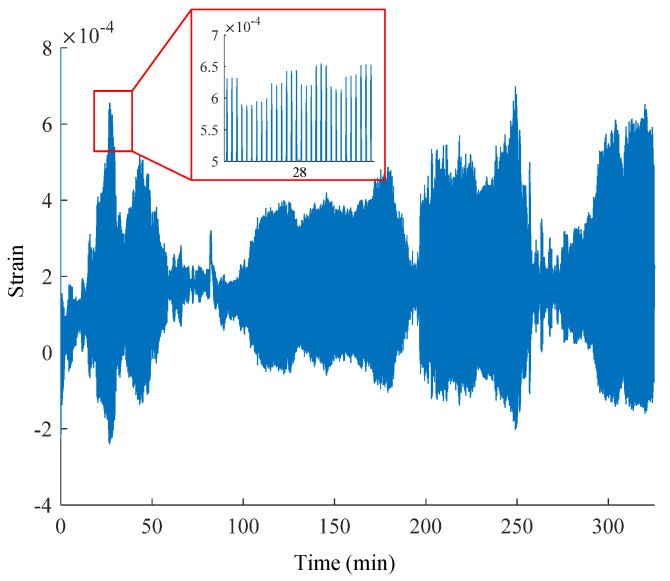
The original strain data.

The strain data is a row vector with dimensions 1 by 19,380,000 and it is shown in Figure 12 (taking strain sensor 1 as an example). The absolute value of the strain in the figure reflects the magnitude of strain, and the sign reflects the direction of the load on the WTB, with tensile strain as positive and compressive strain as negative. According to the alternating changes in the peak and valley values of the strain, it is evident that the strain gauges are repeatedly subjected to tensile and compressive loads as a result of the continuous vibration of the WTB in the flap direction under the linear reciprocating load. The strains in the figure exhibit approximately symmetrical distribution about the baseline, indicating that the flap deformation of the WTB always remains symmetry in experiments. During the initial 20 min, the baseline gradually increased from 0 to about 1.7 × 10^−5^. This phenomenon is not the zero-point drift caused by the measurement system, but the real change of the WTB strain state. The WTB enters the rapid degradation period at the beginning of vibration. The tensile stiffness decreases rapidly and causes the tensile strain to become larger and the compressive strain to become smaller. After about 20 min, the WTB enters the period of stabilization, during which the stiffness remains stable, and the tensile and compressive strains also remain stable. In other words, the change of baseline reflects the fatigue process of the WTB.

Analysis of the strain data in the locally magnified graph around the 28th minute reveals a consistent pattern where every three continuous peaks (flap-cycles) constitute one complete flap-period. The phenomenon aligns with the load design above, wherein the flap-period corresponding to a certain wind speed consists of three flap-cycles.

**Figure 13:**
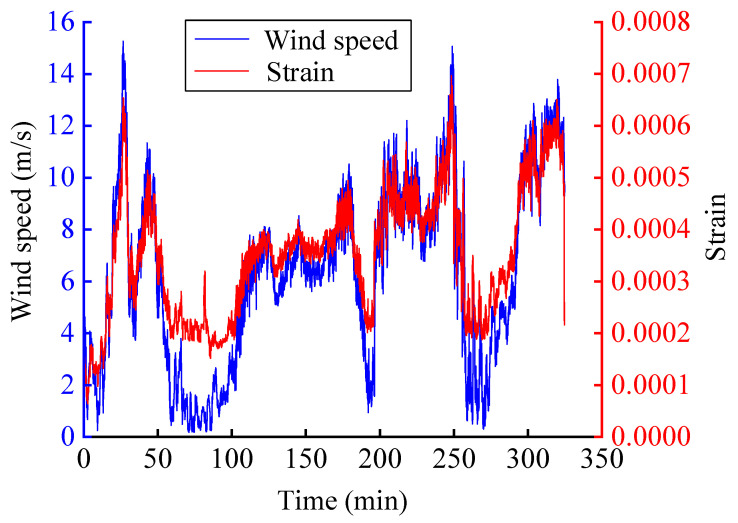
The strain envelope and wind speed time series course.

In order to observe more clearly the process of strain values following the load, the strain data are subjected to envelope analysis and the extracted peak envelope is shown in Figure 13. The horizontal axis of the graph is the time course, the blue line represents the wind speed data, and the red line represents the strain data. It can be clearly found that the strain data show a strong positive correlation with wind speed variations. The maximum peak of wind speed occurs at approximately the 28th and 250th minutes, and accordingly, the strain data similarly reach the maximum values at the 28th and 250th minutes. This consistent temporal alignment is evident both in the global trend analysis and localized short-term observations, confirming that strain fluctuations faithfully track wind speed changes throughout the entire monitoring period. As the wind speed declines to its trough, the resulting load and deformation on the WTB diminish, causing the strain value to decrease correspondingly to a local minimal value. Conversely, when the wind speed rises to its peak, the increased load and deformation strive on the strain value to a local maximum.

In summary, the strain data collected in this experiment are highly fitted to the wind speed. It can be assumed that the collected strain data capture the characteristics of the simulated wind speed. It shows that it is feasible and effective to use this data as a dataset to test the proposed long-time series forecast method under wind load.

## 5. Results and Discussions

### 5.1. Forecast Results Analysis

The first 60% of the strain dataset in time series is employed as the training set and the last 40% serves as the test set. During training the model via the training set, every 100 consecutive data points are taken as a set of inputs, and the 10 consecutive data points following immediately are taken as the corresponding prediction targets. In other words, the length of retrospect window is 100 and the length of forecast window is 10. The Table 2 shows the prediction accuracy for different lengths of retrospect window. The model performs best at the length of 100 and decreases significantly when the length is greater than 200.

Crucially, preserving temporal order is fundamental for time series forecasting. Both input and target vectors must maintain strict chronological alignment of their constituent elements to ensure the integrity of temporal dependencies in the data.

Consistent with the training, the lengths of the retrospect and forecast windows are 100 and 10, respectively, during the testing. In the conventional testing method, each input sequence operates independently, with no temporal dependencies between successive test samples. The evaluation remains unaffected even if the original chronological order of test input sequences is randomized. In practical applications, however, only the elements contained in the first set of inputs are known, while all subsequent inputs represent future unknown states.

Consequently, this research adopts an alternative testing method that more accurately reflects the actual engineering problems, departing from conventional approaches. The testing process iteratively incorporates the forecast values into new test inputs to advance the forecast window stepwise. In this way, the propagation error is cumulative and will have some influence on the subsequent prediction. It is more in line with future unknown problems in actual engineering. Therefore, in the testing phase of this research, only the first set of actual values in the test set are employed as inputs, while the other actual values are only employed as the corresponding labels to evaluate the forecast accuracy.

**Figure 14:**
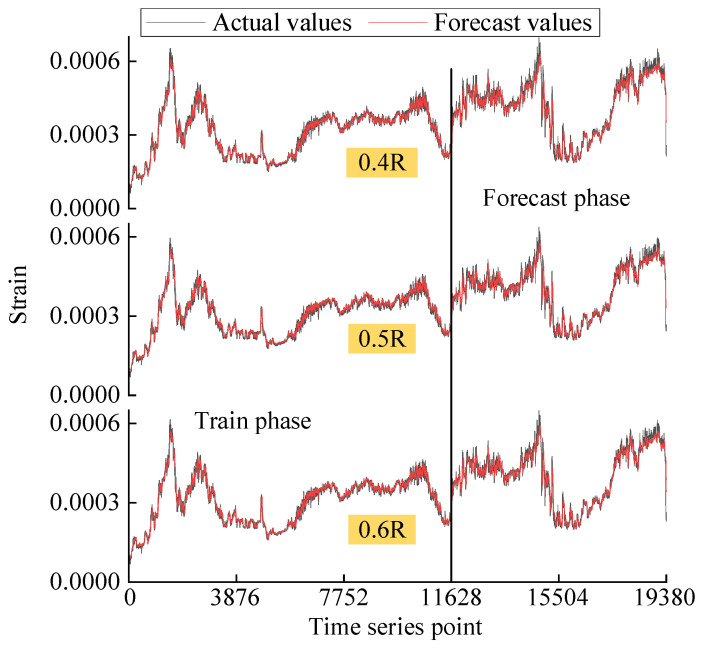
Forecast results of the three datasets.

It is assumed that the strain in the first 60% has already occurred while the strain in the last 40% has not yet occurred, which is unknown future data. The incremental forecast model is trained with the first 60% of strain data to learn the changing regularity of strain. Then, the model forecasts the last 40% of the strain data. The forecast results are shown in Figure 14. The vertical axis of the figure is the strain value and the horizontal axis is the index of the data series point. The results of three different datasets, 0.4R, 0.5R, and 0.6R, are distributed from top to bottom. The black lines are the actual strain values measured from sensors and the red lines are the forecast strain values computed from model. The first 60% is the training phase and the last 40% is the testing phase. It can be found that the three datasets exhibit consistent temporal patterns despite minor variations in strain values. After training, the forecast values of the model fit well with the actual values. Some relatively significant errors occur only at some peaks, valleys where strain values change abruptly.

**Figure 15:**
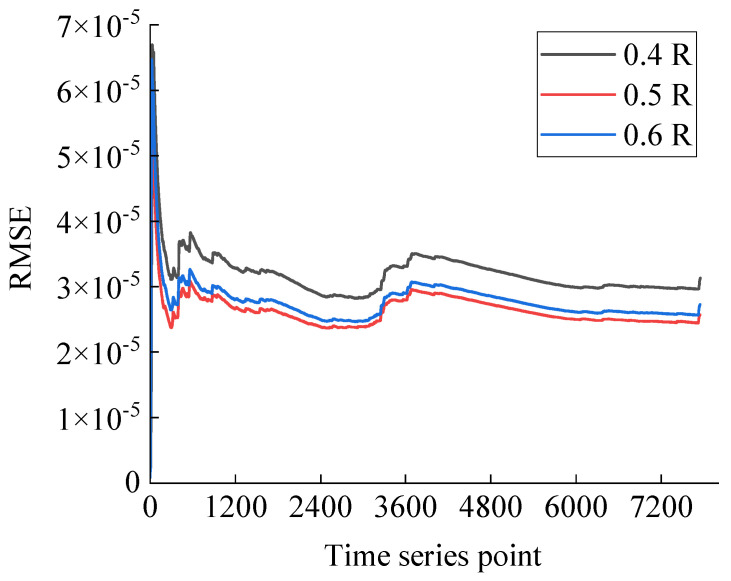
Error dynamics during the forecast.

In order to better analyze the forecasting performance of the model, the root mean square error (RMSE) between the forecast and actual values during the forecast phase is tracked to quantify the forecast accuracy, as shown in Figure 15. The RMSE counts the current moment as well as all previous errors, and is able to reflect the effect on forecast values from accumulation of errors over the time. The RMSE in figure remains at a small value overall, indicating that the model has a high accuracy in prediction. The high RMSE in the short period of time at the beginning is due to the fact that the small amount of data only reflects limited information as a statistical indicator. As the number of forecast values increases, the RMSE decreases rapidly to lower values and is maintained over time. There are two significant bumps which indicate a larger deviation in the current forecast values due to the effect of the errors from the previous forecast values. Larger slopes indicate that the error accumulates rapidly and has a great impact on the subsequent forecast values. The subsequent decrease after the bump is due to the fact that the model detects an anomaly in the error and initiates the incremental learning to dynamic update the parameters. The forecast accuracy of the updated model returns to a higher level, and the RMSE gradually decreases. It is clear that the incremental learning mechanism of the model is able to dynamically update the parameters according to the arrival of new data, and thus maintain a high forecast accuracy over time.

### 5.2. Comparative Analysis

Comparative analysis is an essential part of evaluating the proposed forecast method. According to the operational mechanisms, recurrent neural networks (RNN) and self-attention are the two most dominant classifications in current forecast model research. One of the classical representatives of RNN is Bi-LSTM, while the classical representative of Self-attention is Transformer, whose performance has a significant improvement over RNN. Therefore, the above two models are selected as the reference representatives for RNN and Self-attention, respectively, to compare and discuss the performance with the incremental forecast method proposed in this research.

**Figure 16:**
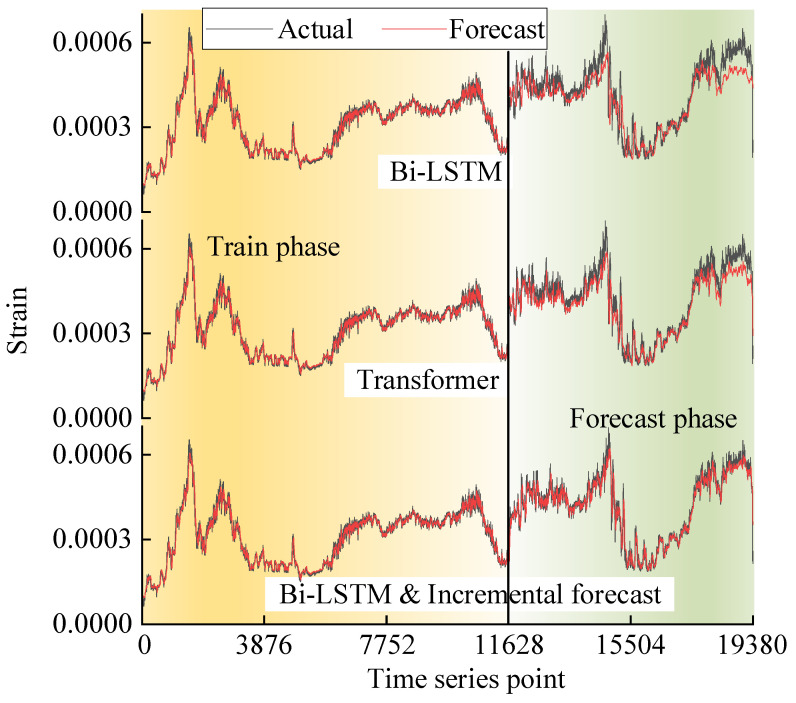
Comparison of different methods for forecast on 0.4R strain data.

Take the strain data on 0.4R as an example; Figure 16 shows the test results of the different methods. In each of these figures, the forecast results of Bi-LSTM, Transformer and incremental forecast methods are in order from the top to the bottom, with the black lines being the actual strain values measured from sensors and the red lines being the forecast strain values computed from model.

It can be seen that the forecast values of the three methods can keep the same change regularity with the actual values. However, the three methods show significantly different performance in the final stage. Since the accumulation of errors in the early stage has a serious impact on the prediction accuracy, it can be clearly observed that both the Bi-LSTM and Transformer show a significant deviation between the forecast and actual values in the final stage. Conversely, the incremental forecast method can effectively eliminate the effect of error accumulation via the error-supervised feedback mechanism. As a result, the incremental forecast method exhibits superior prediction accuracy in the final stage.

In addition, the Bi-LSTM shows a significant lag near the peak mutation. This is due to the inevitable gradient vanishing, which leads to the forgetting of some historical features and short-term memory struggling to respond to the data mutations in time. This phenomenon is significantly improved because the model structure of the Transformer and incremental forecast method can capture historical features better than Bi-LSTM. Moreover, the incremental forecast method outperforms the Transformer.

In this research, the coefficient of determination R^2^, which is widely used to evaluate regression models, is adopted to further quantify the forecast performance of the three methods. When its value is closer to 1, it indicates that the data fit better. Whereas, its value tends to 0 when the model does not capture the characteristics of changes in the data and performs poorly. The formula for R^2^ is as follows:

(20) $$R^{2}=\frac{\sum_{i=1}^{n}{\left(y_{i}-{\hat{y}}_{i}\right)}^{2}}{\sum_{i=1}^{n}{\left(y_{i}-\bar{y}\right)}^{2}}$$

where ${\hat{y}}_{i}$ is the *i*th forecast value, $y_{i}$ is the *i*th actual value and ${\bar{y}}_{i}$ is the mean value of actual values.

**Figure 17:**
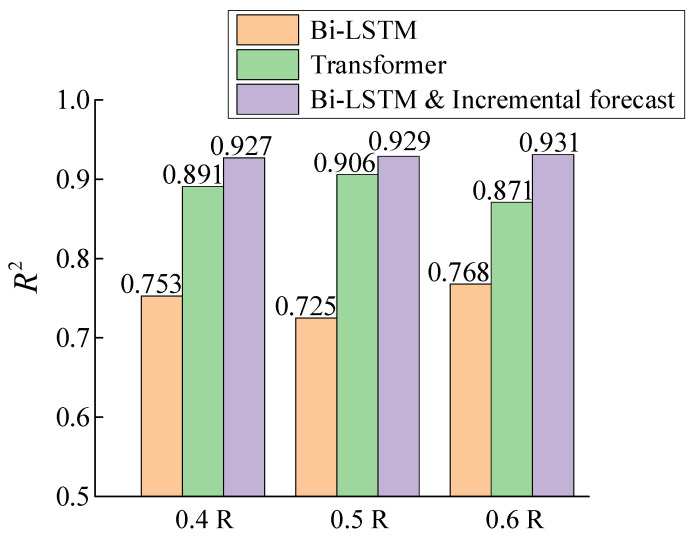
Comparison of different method for forecast on R^2^.

The R^2^ of the three methods on each of the three datasets is shown in Figure 17. It is clearly seen that the incremental forecast outperforms the other two methods on all three datasets, with R^2^ values being 0.929 (mean of 0.4R, 0.5R and 0.6R). Additionally, the R^2^ values of Bi-LSTM and Transformer are 0.749 and 0.889, respectively. According to the above, the forecast accuracy is improved by 24% and 4.6% compared with Bi-LSTM before improvement and Transformer. Although the performance of the Transformer lightly decreases compared to the incremental forecast, it has a significant improvement compared to the Bi-LSTM. The above fully illustrates that although the Bi-LSTM performs the worst among the three methods, the incremental forecast method based on the Bi-LSTM is able to substantially improve the long-time forecast, even surpassing the Transformer. The reason for this phenomenon is the unique self-updating structural designed in the proposed incremental forecast method.

The inference time and memory usage of the above three methods are shown in the Table 3. The performance of the incremental forecast method is obviously optimal.

### 5.3. Ablation Analysis

In order to analyze the preprocessing sensitivity on prediction accuracy, the preprocessed and original data are input into the incremental forecast model under the same model settings, respectively. The R^2^ of the forecast results are shown in the Table 4. Obviously, the preprocessing improves the prediction accuracy by 2.9%.

The K-means clustering during experience replay and the EWC regularization during training are two very important designs for the proposed incremental forecast architecture. In order to validate the contribution of both for the proposed method, an ablation experiment is designed to analyze the effects of K-means clustering and EWC regularization on the performance of proposed method, respectively.

The ablation experiment is designed with four schemes: Scheme 1 is a complete incremental forecast method. Scheme 2 only retains the EWC regularization, and the K-means clustering is instead of the conventional uniform probability of random sampling. Scheme 3, opposite of Scheme 2, retains only the K-means clustering and removes the EWC regularization during training. Scheme 4 further eliminates the K-means clustering on the basis of Scheme 3, i.e., only retains the initial incremental learning process. The three datasets are entered into the model for each of the four schemes described above, with all settings remaining the same as before.

**Figure 18:**
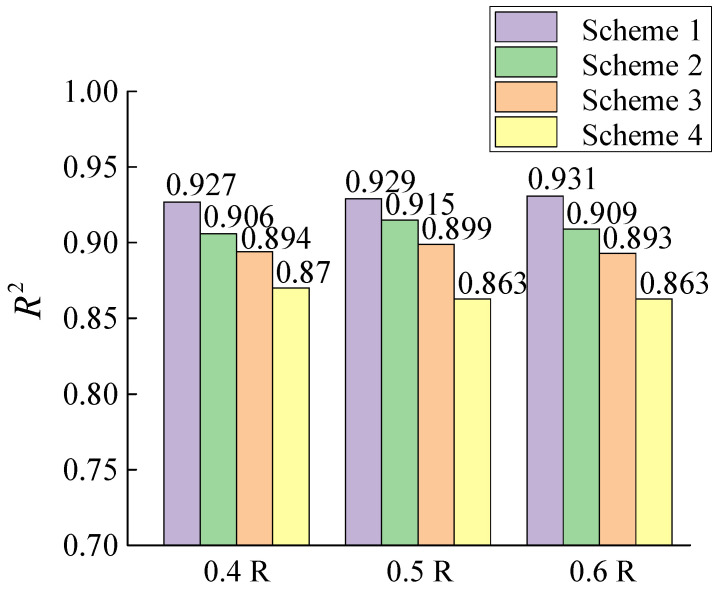
Comparison of different scheme for forecast on R^2^.

The R^2^ of the forecast results for each group of schemes is calculated to evaluate the performance, as shown in Figure 18. Scheme 4 substantially improves the R2 score by almost 10% compared to that without incremental forecast, Bi-LSTM in the above section, demonstrating the importance of the incremental learning structure. However, Scheme 4 performs the worst in ablation comparisons. The fitting effects of Scheme 2 and 3 are relatively close to each other, indicating that the EWC regularization and the K-means clustering, contribute equally to the improvement of forecast accuracy, with the former being slightly higher than the latter. The combined effect of the two will further improve the forecast performance to the level of Scheme 1. All three datasets exhibit the same phenomena.

The error statistics for all forecast results are shown in Table 5. The mean absolute percentage error, MAPE, reflects the overall relative difference between the forecast and actual values. The mean absolute error, MAE, provides the most intuitive view of the average deviation of the forecast results. The root mean square error, RMSE, reflects fluctuations in error and is sensitive to error outliers. The data in Table 3 illustrate again that the forecast accuracy and stability of Scheme 4, Scheme 3, Scheme 2, and Scheme 1 are sequentially increasing, and it validates the effectiveness of the proposed incremental forecast method.

### 5.4. Discussion of the Experiment Limitations

It must be emphasized that the loading methods and the test setup in this research are simplifications. However, the actual operating state of the WTB is very complex. For example, the aeroelastic effect makes load variable even if the wind speed is constant, and the strain will exhibit periodic characteristics under the rotating centrifugal force. In addition, the vibration contains flap-wise, edge-wise and twisting directions. But it is difficult to restore all the states under laboratory conditions. Therefore, this research simplifies the loading methods and only considers the vibration in the flap-wise direction.

## 6. Conclusions

This article conducts a comprehensive study on methods to address the challenge of accurately predicting the long-time health status of a WTB. The research reveals that the accumulation of errors is the primary reason for the suboptimal performance of current popular methods in long-term time series forecasting. Consequently, an incremental forecast method endowed with adaptive updating capabilities is proposed to mitigate the phenomenon of error accumulation, thereby enhancing the accuracy of long-time series forecasting. The method adopts the idea of incremental learning to improve the Bi-LSTM model via the error-supervised feedback structure designed in model. Then, the model is capable of continuously updating the parameters as new data become available, thereby maintaining a high level of accuracy in long-time forecast.

Furthermore, an experimental setup for strain detection on WTBs is constructed in this research. The strain data collected from this experimental platform are utilized to test the accuracy of the proposed incremental forecast method. The results show that the proposed method can maintain high accuracy in accomplishing the long-time series forecast task, with a fitting accuracy of 0.929 to the actual values and an average error below 4.9%. The forecast accuracy is improved by 24% compared with that of Bi-LSTM before improvement, and by 4.6% compared with that of Transformer.

The experimental results demonstrate that the incremental forecast method pro-posed in this research can effectively forecast the long-term strain of WTBs, showing the great potential value for engineering applications. Meanwhile, it provides a novel technical idea and theoretical guidance for the long-time series forecast problem. On this basis, the methodologies of setting thresholds more rationally and evaluating the health status of WTBs based on predicted strain values are very promising for future research. In the meantime, the improvement of the experimental setup to take into account more actual operating conditions is equally exciting.

## Figures and Tables

**Table 1:** The characteristics of the strain sensor.

Wire Grid Size (mm)	Substrate Size (mm)	Resistance (Ω)	Strain Limit	Sensitivity Coefficient
2.0 × 1.0	3.6 × 3.1	350 ± 0.1	2%	2.0 ± 1%

**Table 2:** The sensitivity analysis of window length.

Window Length	50	100	200	300
R^2^	0.897	0.929	0.902	0.838

**Table 3:** The inference time and memory usage.

	Bi-LSTM	Transformer	Incremental Forecast
Inference time	4 ms	15 ms	4 ms
Memory usage	3.2 G	14.9 G	5.2 G

**Table 4:** The R^2^ of result of the preprocessed and original data.

	0.4R	0.5R	0.5R	Mean
Preprocessed	0.927	0.929	0.931	0.929
Original	0.896	0.901	0.903	0.900

**Table 5:** The error statistics of different ablation scheme on each dataset.

Indicators	Dataset	Scheme 1	Scheme 2	Scheme 3	Scheme 4
MAPE	0.4R	5.36%	6.08%	6.38%	7.10%
	0.5R	4.56%	4.76%	5.34%	6.12%
	0.6R	4.77%	5.32%	5.96%	6.61%
MAE	0.4R	2.15 × 10^−5^	2.55 × 10^−5^	2.58 × 10^−5^	2.89 × 10^−5^
	0.5R	1.77 × 10^−5^	1.87 × 10^−5^	2.11 × 10^−5^	2.45 × 10^−5^
	0.6R	1.86 × 10^−5^	2.17 × 10^−5^	2.39 × 10^−5^	2.67 × 10^−5^
RMSE	0.4R	3.13 × 10^−5^	3.56 × 10^−5^	3.77 × 10^−5^	4.18 × 10^−5^
	0.5R	2.57 × 10^−5^	2.72 × 10^−5^	3.05 × 10^−5^	3.57 × 10^−5^
	0.6R	2.73 × 10^−5^	3.13 × 10^−5^	3.38 × 10^−5^	3.83 × 10^−5^

## Data Availability

The raw data supporting the conclusions of this article will be made available by the authors on request.
